# RNAi-Mediated Knockdown of the Sodium Channel Auxiliary Subunit *TipE* Increases Pyrethroid-Associated Mortality and Reveals Metabolic Transcriptional Responses in *Megalurothrips usitatus*

**DOI:** 10.3390/genes17080854

**Published:** 2026-07-24

**Authors:** Likui Wang, Siqing Zhang, Linlin Yuan, Weiwei Wu, Huihui Wu, Zhengke Peng, Pei Liang, Kun Zhang, Shaoying Wu

**Affiliations:** 1 Sanya Institute, China Agricultural University, Sanya 572024, China; 19937157228@163.com (L.W.);; 2 School of Breeding and Multiplication (Sanya Institute of Breeding and Multiplication), Hainan University, Sanya 572024, China; zsq010416@163.com (S.Z.);; 3 School of Tropical Agriculture and Forestry, Hainan University, Danzhou 571700, China; 4 School of Tropical Fruits and Vegetables, Hainan University, Baoting 572300, China; 5 Institute of Plant Protection, Guangdong Academy of Agricultural Sciences, Guangzhou 510640, China

**Keywords:** *Megalurothrips usitatus*, pyrethroid susceptibility, sodium-channel auxiliary subunit, *TipE*, RNA interference, transcriptome

## Abstract

**Background**: The bean flower thrips, *Megalurothrips usitatus*, is a destructive agricultural pest with increasing resistance to pyrethroid insecticides. Most mechanistic studies emphasize mutations in the pore-forming voltage-gated sodium channels α-subunit, whereas the contribution of insect-specific sodium-channel auxiliary subunits remains unclear. **Methods**: We used dsRNA feeding, pyrethroid bioassays, RNA sequencing, and RT-qPCR validation to evaluate the role of temperature-induced paralytic E (*TipE*) in *M. usitatus*. **Results**: The optimized RNAi condition was 400 μg/mL of *dsTipE* for 48 h, which reduced *TipE* expression by 32.55%. *TipE* knockdown increased adult mortality after exposure to λ-cyhalothrin and permethrin, indicating increased pyrethroid-associated susceptibility under laboratory conditions. Transcriptome sequencing identified 205 differentially expressed genes, with enrichment in metabolism-related pathways. RT-qPCR confirmed reduced expression of *CYP307a1*, an ecdysteroid-biosynthesis-related P450 gene, and *SCD*, a lipid-metabolism gene, while Na_v_ α-subunit transcript abundance did not change significantly. **Conclusions**: These results identify *TipE* as an auxiliary-subunit factor associated with pyrethroid susceptibility in *M. usitatus* and provide a transcriptome-based working model linking sodium-channel auxiliary regulation with endocrine- and lipid-metabolism-related responses. This study expands the candidate space for pyrethroid resistance research beyond Na_v_ α-subunit mutations, although the causal roles of *CYP307a1* and *SCD* require further validation.

## 1. Introduction

The bean flower thrips, *Megalurothrips usitatus* (Bagnall), is a major pest of cowpea and other leguminous vegetables in tropical and subtropical production systems [1,2]. Hainan Province provides a warm and humid environment that is highly suitable for the development, reproduction, and dispersal of *M. usitatus* [2]. Under these conditions, populations can occur throughout the year, with no clear overwintering diapause, rapid generation turnover, and strong overlap among developmental stages [1]. Such population characteristics make field infestations difficult to control once outbreaks begin and can directly reduce flower retention, pod development, yield, and commercial quality in cowpea production [2]. Current management still depends largely on chemical insecticides. Pyrethroids, including λ-cyhalothrin, permethrin, and bifenthrin, remain important field options because of their rapid knockdown activity, relatively high selectivity against target pests, and operational convenience [3]. However, repeated applications under intensive vegetable production have promoted resistance development in *M. usitatus* populations. This reduces control efficacy, increases control costs, and raises the risk of excessive pesticide residues and environmental pressure [4]. Recent resistance-monitoring and sodium-channel studies in *M. usitatus* further indicate that local pyrethroid resistance is not only a field-management problem but also a molecular-evolution problem involving target-site variation and additional regulatory mechanisms [5,6,7]. Therefore, clarifying the molecular determinants of pyrethroid susceptibility in *M. usitatus* is important for resistance monitoring, resistance-management planning, and the development of more selective control strategies.

Voltage-gated sodium channels (Na_v_) are the primary molecular targets of pyrethroids in insects and are central to knockdown resistance (kdr), one of the best-characterized mechanisms of pyrethroid resistance [5]. In *M. usitatus*, field-associated Na_v_ mutations, including T929I and K1774N, have been functionally linked to reduced pyrethroid sensitivity [6], and sodium-channel mutation screening in Hainan populations further supports the importance of target-site variation in local resistance evolution [7]. Sodium-channel mutations reported in other insects, such as V410L in *Aedes aegypti*, also demonstrate the broad contribution of Na_v_ variation to pyrethroid resistance [8]. Mechanistic studies have shown that pyrethroids bind to sodium channels in nerve membranes, prolong channel opening, delay deactivation and inactivation, and ultimately disrupt normal nerve excitation, producing the characteristic knockdown phenotype [9,10]. Nevertheless, pyrethroid resistance is rarely explained by a single mutation alone. The functional sensitivity of insect sodium channels can be affected by channel expression abundance, alternative splicing, RNA editing, auxiliary subunits, membrane environment, and interactions with detoxification or metabolic pathways [11,12]. These regulatory layers suggest that genes associated with sodium-channel function, even when they are not the pore-forming α subunit itself, may contribute to differences in pyrethroid susceptibility among pest populations.

Temperature-induced paralytic E (*TipE*) is an insect-specific sodium-channel auxiliary subunit first characterized in *Drosophila*, where it strongly enhances the functional expression of the *para* sodium-channel α subunit and is required for normal neuronal excitability [13,14]. Intron retention and transcript variation in insect *TipE*-like genes can alter sodium-channel function and drug sensitivity, showing that post-transcriptional regulation of auxiliary subunits may influence insect responses to neurotoxic compounds [15]. Comparative evolutionary analyses indicate that *TipE* and *TipE*-homologous (*TEH*) proteins form a conserved insect auxiliary-subunit family with features distinct from vertebrate sodium-channel β subunits [16]. Functional analyses in the house fly and related studies of *Drosophila* homologs further indicate that *TipE*/*TEH* proteins can modulate channel expression, current density, gating properties, membrane localization, and pharmacological sensitivity [17,18]. Compared with the extensive literature on Na_v_ α-subunit mutations, however, the roles of sodium-channel auxiliary subunits in insecticide resistance remain less well defined. This gap is especially important for small agricultural pests such as *M. usitatus*, in which resistance monitoring has focused mainly on insecticide bioassays and Na_v_ mutation detection rather than auxiliary-channel regulation. *TipE* may affect pyrethroid response through direct effects on sodium-channel function or through indirect physiological changes caused by altered neural excitability. At present, the contribution of *TipE* to pyrethroid susceptibility in *M. usitatus* has not been experimentally clarified, and its possible links with downstream transcriptional or metabolic changes remain unknown.

RNA interference (RNAi), mediated by double-stranded RNA (dsRNA), provides an effective approach for functional analysis in non-model insects and has become an important tool in insect toxicology and pest management research [19,20,21,22]. Because RNAi can reduce target-gene expression without the need for stable transformation, it is particularly useful for testing candidate genes in small pest species where genetic tools are still limited. Early plant-mediated and diet-delivered RNAi studies demonstrated that dsRNA could suppress essential or detoxification-related genes and reduce pest survival or stress tolerance, supporting the feasibility of RNAi-based control strategies [23,24]. RNAi has since been used to evaluate genes encoding neural targets, ion channels, cuticle- or chitin-related enzymes, antioxidant enzymes, and detoxification proteins in multiple pest species [21,22,25,26,27,28,29]. For example, silencing Na_v_ genes reduced survival in *Tribolium castaneum* and *Myzus persicae* [25,27], while RNAi targeting other essential genes decreased longevity, fecundity, or developmental success in *Aphis gossypii* and *Spodoptera litura* [28,29]. In thrips, advances in dsRNA delivery and formulation also indicate that RNAi can be adapted for small-bodied sucking or rasping-feeding pests [26]. These studies provide both conceptual and methodological support for testing whether *TipE* knockdown can change pyrethroid susceptibility in *M. usitatus*.

In addition to target-site mechanisms, metabolic regulation is a major component of insecticide resistance. Cytochrome P450 monooxygenases participate in xenobiotic detoxification and are frequently associated with insecticide resistance in insects [30,31]. Changes in detoxification genes can also interact with broader physiological processes, including development, oxidative stress, hormone biosynthesis, and lipid metabolism [31,32]. Lipid metabolic enzymes such as stearoyl-CoA desaturases (*SCD*) are essential for membrane composition, development, reproduction, and stress tolerance in insects and other animals [33,34,35]. Together, these observations identify an unresolved question: can perturbation of an insect-specific sodium-channel auxiliary subunit change pyrethroid susceptibility and, at the same time, reshape metabolic transcriptional programs that may influence the phenotype? Addressing this question would extend pyrethroid-resistance research in *M. usitatus* from mutation detection in the Na_v_ α-subunit to functional testing of auxiliary-channel regulation. In this study, we combined dsRNA feeding, pyrethroid bioassays, transcriptome sequencing, and reverse-transcription quantitative PCR (RT-qPCR) validation to evaluate the function of *TipE* in *M. usitatus*. By linking a measurable susceptibility phenotype with downstream candidate genes, especially cytochrome P450 *307a1* (*CYP307a1*) and *SCD*, we aimed to provide a testable framework for future mechanistic studies and resistance-management target discovery.

## 2. Materials and Methods

### 2.1. Test Insects

The *M. usitatus* used in the experiments were obtained from a pyrethroid-susceptible laboratory colony that was established from a field population collected at the Hainan University Agricultural Science Base in 2017 and has been continuously reared at the Laboratory of Green Prevention and Control of Tropical Pests, Hainan University. The colony was maintained on fresh cowpea pods under controlled laboratory conditions at 26 ± 1 °C, 65 ± 5% relative humidity, and a photoperiod of 16 h light and 8 h darkness, following previously described cowpea rearing methods for *M. usitatus* [36,37]. Fresh cowpea pods were surface-disinfected with 0.5% sodium hypochlorite, rinsed with distilled water, and air-dried before use. The pods were replaced every three days to prevent mold growth and food deterioration.

During colony maintenance, adults were kept together with eggs and larvae on cowpea pods and were not separated by developmental stage. Because *M. usitatus* females lay eggs into the superficial tissues of cowpea pods, larvae were transferred only after egg hatching. When cowpea pods were replaced, newly hatched larvae remaining on the old pods were gently transferred to fresh cowpea pods in the rearing bottles using a fine brush. Healthy and active adults of comparable body size were selected for RNAi feeding and bioassays.

### 2.2. dsRNA Synthesis and Feeding-Mediated TipE RNAi

The sequence of *M. usitatus TipE* used for dsRNA design was obtained from the chromosome-level genome assembly of *M. usitatus* reported by Ma et al. [38] (GenBank accession JAPTSV000000000; transcript Mu01G011700.1; gene_id ONE63_000579). The double-stranded enhanced green fluorescent protein RNA (ds*EGFP*) negative-control sequence was based on GenBank accession MW246027.1. RNA interference primers were designed based on the *TipE* sequence using the DSIR online tool (http://biodev.extra.cea.fr/DSIR/DSIR.html; accessed on 10 July 2024) [39]. Subsequently, double-stranded RNA (dsRNA) was synthesized in vitro using the T7 High Yield RNA Transcription Kit (Vazyme, Nanjing, China). The in vitro-synthesized double-stranded RNA targeting *TipE* (ds*TipE*) was diluted to twice the target concentration using RNase-free water, and then thoroughly mixed with an equal volume of 20% sucrose solution to prepare the final feeding working solution containing the target concentration of ds*TipE*. To determine the optimal dsRNA feeding concentration, *M. usitatus* adults were fed ds*TipE* at final concentrations of 100, 200, 300, 400, and 500 μg/mL for 48 h, with ds*EGFP* used as the negative control. To determine the optimal feeding duration, adults were fed 400 μg/mL of ds*TipE* for 12, 24, 36, 48, and 60 h.

Feeding-mediated RNAi was performed using a double-layer Parafilm membrane system adapted from previously reported dietary or membrane-feeding approaches for thrips [40,41]. Transparent glass tubes (6 cm in height and 4 cm in inner diameter) were utilized as feeding chambers. The feeding membrane was prepared using Parafilm sealing film (Amcor, Zurich, Switzerland), a semi-transparent thermoplastic sealing film. A piece of Parafilm was cut to cover the opening of the glass tube and evenly stretched in both longitudinal and transverse directions until it became thin and taut. The stretched film was then fixed over one open end of the glass tube. A 300 μL aliquot of the prepared feeding solution was placed onto the center of the membrane, and a second stretched Parafilm layer was immediately placed over the droplet. The edges of the two Parafilm layers were gently pressed together to seal the feeding solution between the membranes and to reduce evaporation and contamination. Healthy and active *M. usitatus* adults with uniform age were selected. To enhance feeding efficiency, the insects were subjected to a 3 h starvation treatment prior to introduction. Subsequently, the insects were transferred into the glass tubes using an aspirator, with 50 adults per tube. Once all test insects were introduced, the bottom opening of the glass tube was sealed and secured with highly breathable rice paper, and the tubes were placed in a cool, shaded indoor environment for rearing.

### 2.3. RT-qPCR Quantification of Gene Expression

Total RNA from *M. usitatus* was extracted using the TRIzol reagent method. Using the extracted RNA as a template, complementary DNA (cDNA) was synthesized via reverse transcription. Specifically, the PrimeScript™ RT reagent Kit with gDNA Eraser (TaKaRa, Kyoto, Japan) was utilized to synthesize cDNA for RT-qPCR. The RT-qPCR experiments for assessing the relative expression of the *TipE* subunit were performed using the TB Green^®^ Premix Ex Taq™ kit (TaKaRa, Kyoto, Japan).

For RT-qPCR validation, the transcript identifiers of the analyzed genes were as follows: *SCD*, Mu13G009390.1 (gene_id:ONE63_003354), *CYP307a1*, Mu11G003590.1 (gene_id:ONE63_001747) and Na_v_, GenBank accession MZ043856. Ribosomal protein L (*RPL*, transcript Mu10G010360.1; gene_id ONE63_001519) was used as the internal reference gene for RT-qPCR normalization. This gene was selected according to a reference-gene validation study in *M. usitatus*, in which *RPL* showed stable expression across tested conditions [42]. Primer specificity was confirmed by melting-curve analysis, and amplification efficiency was checked before relative-expression analysis. For each treatment, three independent biological replicates were prepared, with each biological replicate consisting of 50 pooled *M. usitatus* adults. Each biological replicate was analyzed with three technical qPCR replicates. Relative expression levels were calculated using the 2^−ΔΔCt^ method [43].

### 2.4. Pyrethroid Bioassays After RNAi Treatment

A modified leaf-tube residual film method was employed to conduct bioassays of pyrethroid insecticides on the laboratory population of *M. usitatus* [7,44]. Technical-grade permethrin (95% active ingredient; Shanghai Kelin Biotechnology Co., Ltd., Shanghai, China) and technical-grade λ-cyhalothrin (97% active ingredient; Bide Pharmatech Ltd., Shanghai, China) were used in the bioassays. Both insecticides were technical-grade active ingredients rather than formulated commercial products. Stock solutions were prepared in acetone on an active-ingredient basis. The amount of each technical-grade insecticide used for stock preparation was calculated according to the target stock concentration and corrected for purity using the following formula: mass of technical material = target mass of active ingredient/purity. These stock solutions were then serially diluted with a 0.1% Triton X-100 solution to generate 5 to 6 gradient concentrations, and the prepared solutions were stored in 50 mL tubes for later use. Fresh cowpea pods were cut into uniform small pieces (approximately 1 cm in length) and soaked in the insecticide solutions of different concentrations for 5 min. After removal, they were naturally air-dried at room temperature. The inner walls of 1.5 mL centrifuge tubes were completely coated by immersing them in the corresponding concentrations of the insecticide solutions for 1–2 min. The excess liquid was poured off, and the tubes were placed in a well-ventilated area to air-dry naturally. The treated cowpea pieces were then placed into the dried centrifuge tubes, and 30 adult *M. usitatus* of uniform age were introduced into each tube. Three replicates were established for each concentration, alongside a solvent control group treated solely with 0.1% Triton X-100. Immediately after handling, mortality attributable to mechanical damage was confirmed to be below 5% in all groups, and affected individuals were excluded from the subsequent 48 h mortality analysis. The total number of dead insects was assessed again at 48 h post-treatment. Mortality was corrected using Abbott’s formula when control mortality was between 5% and 20% [45]. Individuals were considered dead when they failed to move after gentle stimulation with a fine brush.

### 2.5. RNA-Seq Sample Collection and Sequencing

Treatments were performed based on the optimal dsRNA feeding conditions determined from the RNAi experiments. The experiment included a ds*TipE* treatment group (interference group) and a ds*EGFP* control group (negative control group), with three biological replicates per group. At 48 h post-interference, surviving *M. usitatus* adult samples from each group were collected and immediately flash-frozen in liquid nitrogen. Following RNA extraction, the concentration and purity of the RNA were measured using a spectrophotometer, and RNA integrity was assessed via agarose gel electrophoresis. Upon passing quality inspection, the RNA samples were shipped on dry ice to Tsingke Biotechnology Co., Ltd. (Beijing, China) for cDNA library construction and transcriptome sequencing. Paired-end sequencing was conducted on the BGI DNBSEQ platform to generate raw sequencing data. Clean reads were analyzed using the chromosome-level genome assembly of *M. usitatus* reported by Ma et al. [38] as the reference genome (GenBank accession JAPTSV000000000).

### 2.6. Transcriptome Data Analysis and DEG Screening

The raw transcriptome data were filtered using fastp software v1.1.0 [46] to remove adapter sequences, reads with a high proportion of unknown nucleotides (N), and low-quality reads. The filtered data were then subjected to quality assessment using FastQC software v0.1.2 [47], where metrics including Q20, Q30, and GC content were evaluated to ensure sequencing quality and obtain high-quality clean reads. Principal component analysis (PCA) was used to evaluate sample reproducibility. KEGG pathway enrichment analysis was performed using clusterProfiler software v4.1.1. Differential expression analysis between the two sample groups was performed using DESeq2 software v3.12 [48]. Differentially expressed genes (DEGs) were screened using |log2FoldChange| ≥ 1 and adjusted *p*-value ≤ 0.05 [49].

### 2.7. Statistical Analysis

One-way analysis of variance (ANOVA) was performed using IBM SPSS Statistics 24, followed by Tukey’s test to compare differences among groups (*p* < 0.05). A two-tailed Pearson test was utilized for correlation analysis, and a hypergeometric distribution test was employed for the KEGG pathway enrichment analysis. Data organization was performed using Microsoft Excel. Bioassay data were processed using Polo software v2.0, where probit analysis was conducted to calculate the LC_50_ values, 95% confidence intervals, regression equation slopes, degrees of freedom, and chi-square values. All bar charts, point-and-line plots, volcano plots of DEGs, PCA plots, clustered heatmaps, and KEGG enrichment bar charts presented in this study were generated using GraphPad Prism 10 software.

## 3. Results

### 3.1. Optimization of dsTipE-Mediated RNAi Conditions

**Figure 1:**
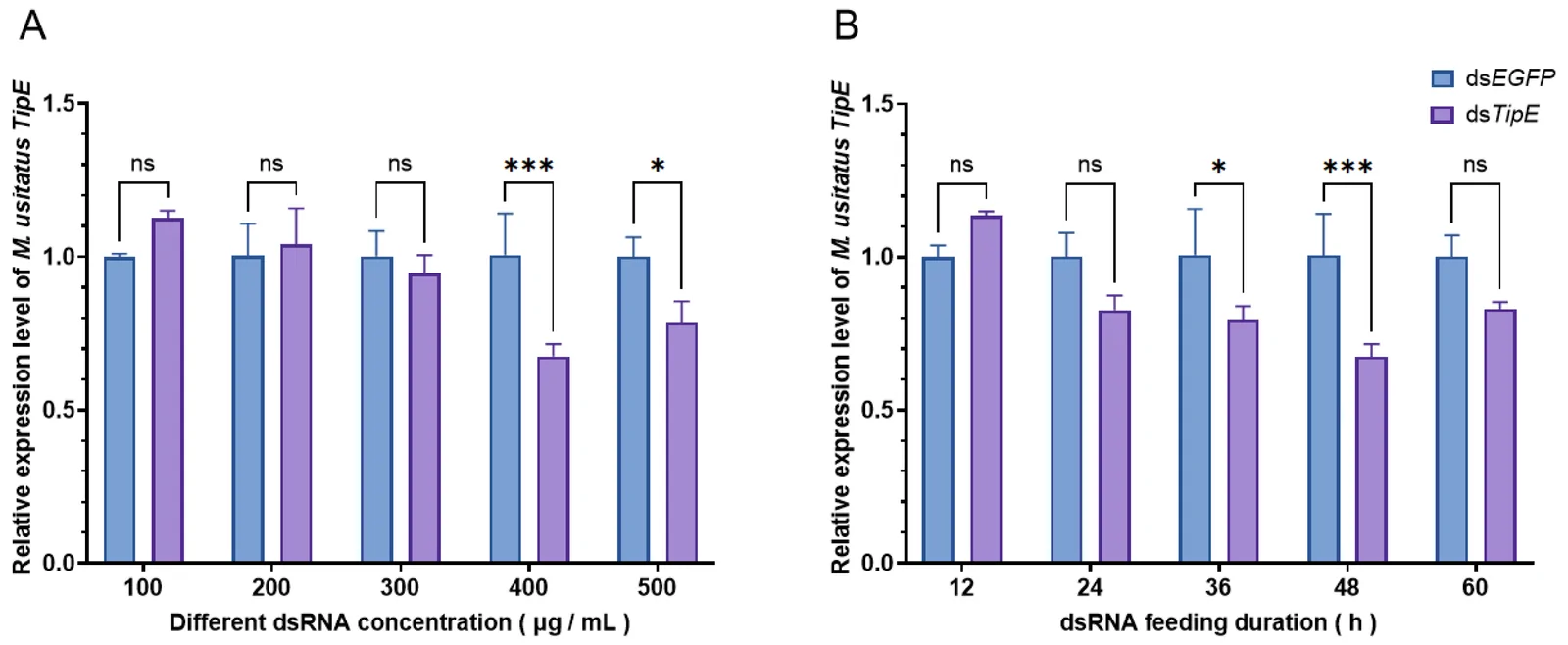
Screening of Optimal Interference Conditions for *TipE* Subunit in *M. usitatus*. (**A**) Changes in relative expression levels of *TipE* subunit under different dsRNA concentrations at a fixed interference time (48 h). (**B**) Changes in relative expression levels of *TipE* subunit under different interference times at a fixed dsRNA concentration (400 μg/mL). For the significance analysis by Tukey’s test, * indicates a significant difference at the 0.05 level (*p* < 0.05), *** indicates a highly significant difference at the 0.001 level (*p* < 0.001), and ns indicates no significant difference (*p* > 0.05).

To determine the optimal dsRNA feeding conditions targeting the *TipE* subunit of *M. usitatus*, this study established different concentration and time gradients for ds*TipE* interference. Feeding with ds*EGFP* served as the negative control, and the relative expression level of the *TipE* subunit in *M. usitatus* was measured using quantitative real-time PCR (Figure 1). Under a fixed interference time of 48 h, the relative expression level of the *TipE* subunit varied with the concentration of ds*TipE*. Specifically, within the ds*TipE* concentration range of 100 to 400 μg/mL, the relative expression level of the *TipE* subunit decreased as the concentration increased. At 400 μg/mL, the relative expression reached its lowest point, which was significantly lower than that of the negative control (*p* < 0.001). However, as the concentration was further elevated (beyond 400 μg/mL), the relative expression level of the *TipE* subunit began to rise again. Furthermore, under a fixed ds*TipE* concentration of 400 μg/mL, five different interference durations were evaluated. Throughout the interference period from 12 to 48 h, the relative expression level of the *TipE* subunit exhibited an overall downward trend. At the 48 h time point, the relative expression level of the *TipE* subunit was at its minimum (67.45%), significantly lower than that of the negative control (*p* < 0.001). Conversely, there was no significant difference in the relative expression level of the *TipE* subunit between the 12 h or 24 h treatment groups and the negative control (*p* > 0.05), indicating that insufficient interference time results in a negligible gene silencing effect. Overall, these results demonstrate that in the RNA interference assay, treating with 400 μg/mL ds*TipE* for 48 h achieves the maximum knockdown efficiency of 32.55%.

### 3.2. TipE Knockdown Increases Pyrethroid-Associated Mortality

To examine whether *TipE* knockdown affects pyrethroid-associated mortality in *M. usitatus*, this study established three treatment groups based on the previously determined optimal RNA interference conditions: feeding with a 20% sucrose solution as the blank control, feeding with ds*EGFP* (targeting a non-specific gene) as the negative control, and feeding with ds*TipE* as the treatment group. The treatment duration for all groups was 48 h, with both ds*EGFP* and ds*TipE* concentrations set at 400 μg/mL. Immediately after the treatment, surviving individuals from each group were collected to conduct bioassays using two pyrethroid insecticides (λ-cyhalothrin and permethrin). Three concentration gradients (625 mg/L, 1250 mg/L, and 2500 mg/L) were established for each insecticide, with three biological replicates per concentration. Handling-related mortality immediately after transfer was below 5% in all treatment and control groups. Pyrethroid-associated mortality was subsequently assessed at 48 h post-treatment.

**Figure 2:**
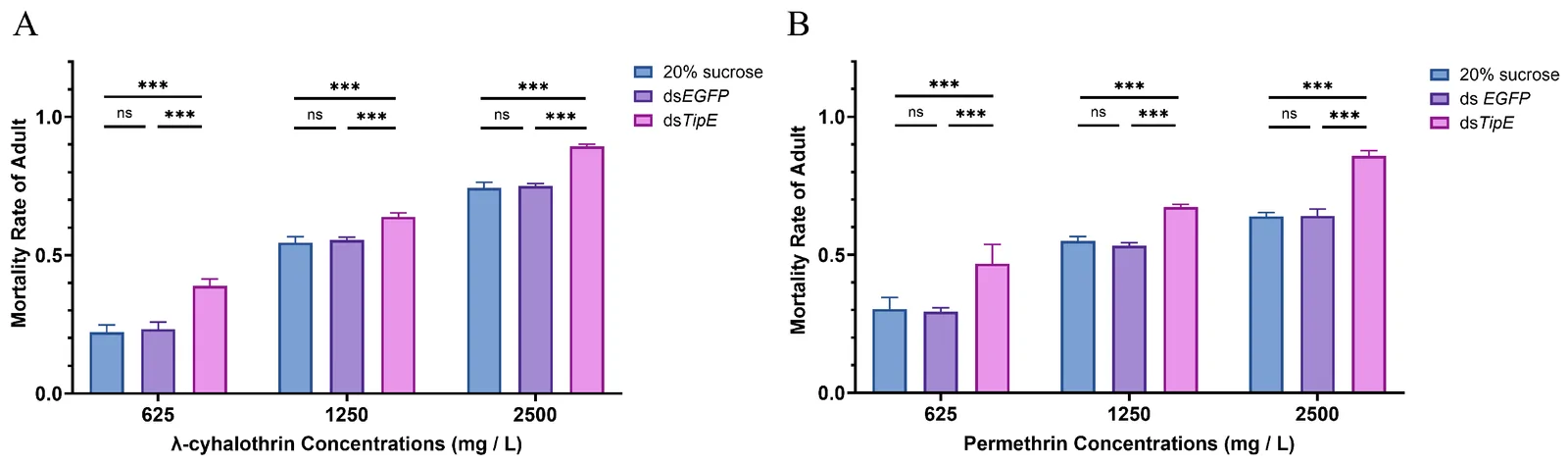
Mortality rates of *M. usitatus* adults treated with different concentrations of pyrethroid insecticides after *TipE* subunit silencing. (**A**) λ-cyhalothrin. (**B**) Permethrin. For the significance analysis by Tukey’s test, *** indicates a highly significant difference at the 0.001 level (*p* < 0.001), and ns indicates no significant difference (*p* > 0.05).

The bioassay results (Figure 2) revealed that as the concentration of the pyrethroid insecticides increased, the mortality rates of *M. usitatus* adults across all three treatment groups exhibited an upward trend. At all treated concentrations, there was no significant difference in mortality between the 20% sucrose solution group and the ds*EGFP* group (*p* > 0.05), indicating that feeding ds*EGFP* did not affect adult mortality after pyrethroid exposure. Conversely, compared with the ds*EGFP* group, adult mortality after exposure to both pyrethroid insecticides was significantly increased in the ds*TipE* treatment group (*p* < 0.05). Specifically, following treatments with 625 mg/L, 1250 mg/L, and 2500 mg/L λ-cyhalothrin, the mortality rates of *M. usitatus* adults increased by 15.64%, 8.36%, and 14.40%, respectively. Similarly, following treatments with 625 mg/L, 1250 mg/L, and 2500 mg/L permethrin, the mortality rates increased by 17.44%, 13.96%, and 21.72%, respectively. These findings demonstrate that silencing the *TipE* subunit leads to an increase in *M. usitatus* mortality, indicating that the *TipE* subunit is one of the key factors involved in pyrethroid resistance.

### 3.3. RNA-Seq Quality Control and Sample Reproducibility

**Figure 3:**
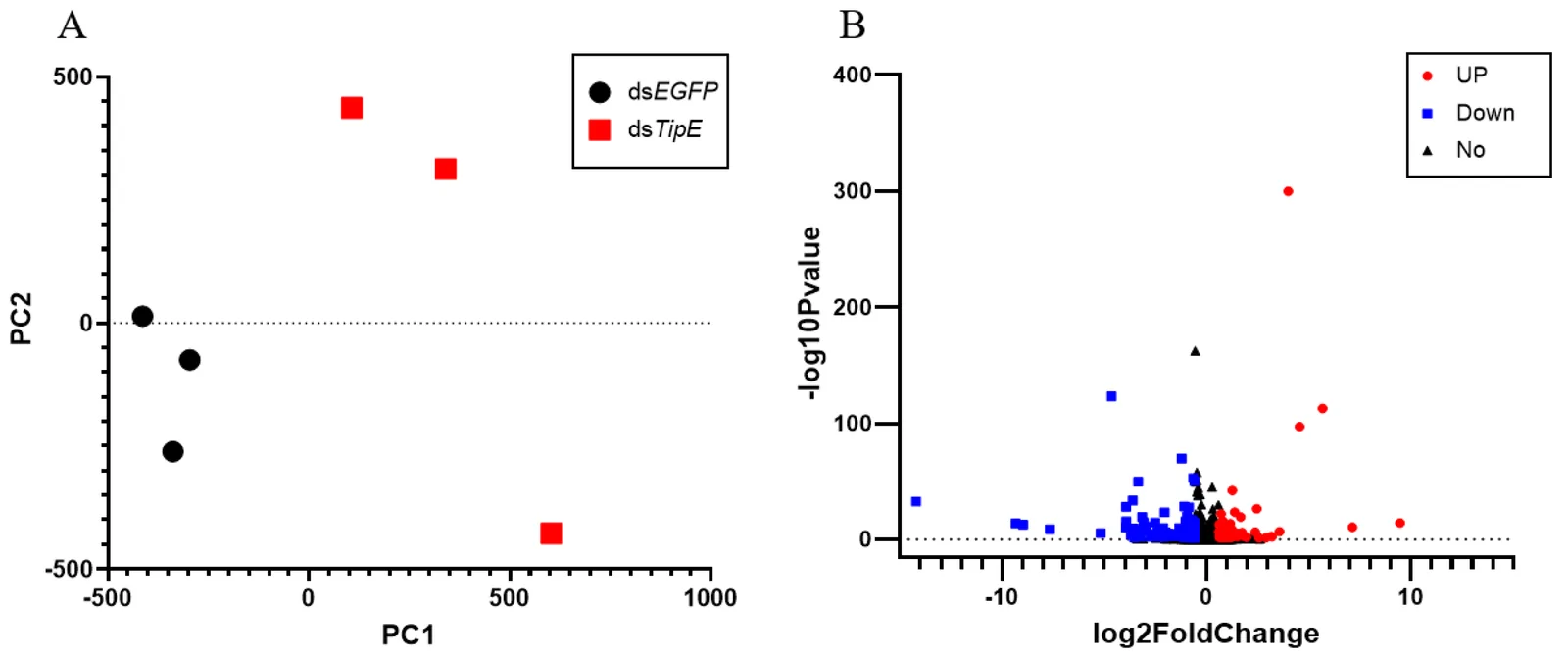
Transcriptome PCA and DEG volcano plot after *TipE* silencing. (**A**) PCA of transcriptome samples. (**B**) Volcano plot of DEGs between *dsTipE* and *dsEGFP*.

The output and quality statistics of the transcriptome sequencing data are summarized in Table 1. After rigorous filtering of the raw reads, the number of high-quality clean reads obtained for each sample exceeded 40 million, with effective rates consistently above 96.6%. The Q20 and Q30 ratios for all samples reached over 95.9% and 93.2%, respectively. Furthermore, the GC content was stably distributed between 42.7% and 43.11%, meeting the requirements for downstream bioinformatic analysis. To evaluate the biological reproducibility and inter-group differences in the samples, Principal Component Analysis (PCA) was performed based on gene expression levels. As shown in Figure 3A, the first principal component (PC1) and the second principal component (PC2) explained 45.52% and 28.72% of the total variance, respectively. In the PCA spatial distribution plot, the three biological replicates from both the *dsEGFP* control group and the *dsTipE* treatment group exhibited tight clustering, indicating highly consistent expression patterns among replicates and excellent intra-group biological reproducibility. Meanwhile, a distinct spatial separation was observed between the treatment and control groups along the PC1 axis, demonstrating that *TipE* subunit silencing significantly altered the transcriptomic expression profile of *M. usitatus*. The clear differentiation between the two groups indicates a high degree of distinguishability.

### 3.4. TipE Knockdown Induces DEGs Enriched in Lipid-Metabolism Pathways

**Figure 4:**
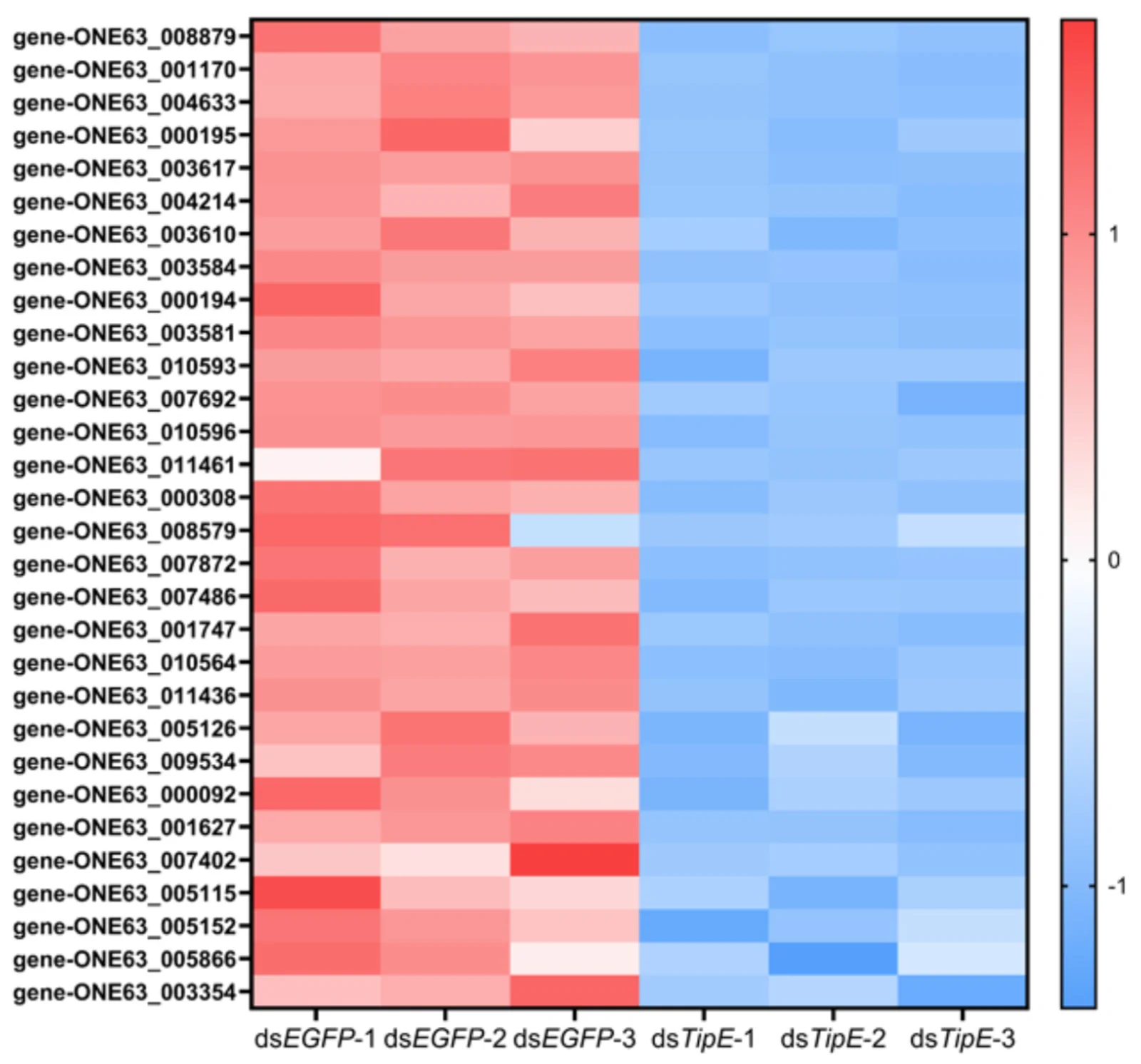
Heatmap of the top 30 significantly downregulated DEGs after *TipE* subunit silencing. Note: Colors represent row-scaled Z-scores of relative expression levels (red: high expression; blue: low expression).

Statistical analysis revealed that, compared to the *dsEGFP* control group, a total of 205 differentially expressed genes (DEGs) were identified in the *dsTipE* treatment group, while the expression of 10,126 genes remained unchanged. As depicted in Figure 3B, the distribution of these DEGs showed an asymmetric pattern, comprising 71 up-regulated genes and 134 down-regulated genes. These results indicate that *TipE* silencing was associated with broad transcript-level changes in *M. usitatus*. To identify genes with strong down-regulation after *TipE* knockdown, we screened the top 30 most significantly down-regulated DEGs and generated a clustered heatmap. The results (Figure 4) showed that these genes had relatively high expression in the *dsEGFP* control group but were consistently reduced in the *dsTipE* treatment group. This pattern suggests that these genes may participate in transcriptional responses associated with *TipE* knockdown and may provide candidate links between *TipE* silencing and the observed increase in pyrethroid-associated mortality.

**Figure 5:**
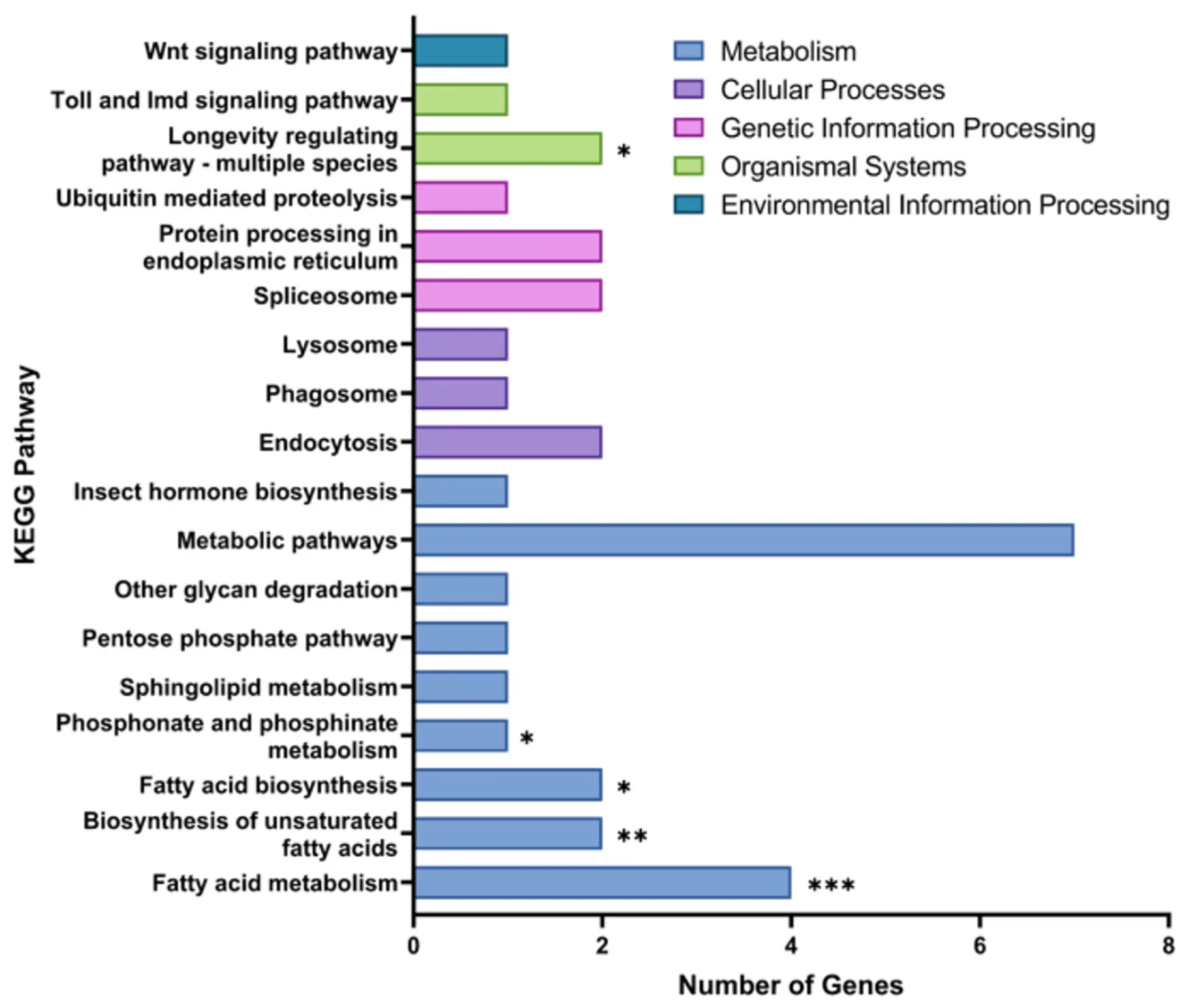
KEGG pathway enrichment analysis of differentially expressed genes (DEGs) in *M. usitatus* after *TipE* subunit silencing. Hypergeometric distribution test was used for KEGG pathway enrichment analysis. * indicates significant enrichment at the 0.05 level (*p* < 0.05), ** indicates very significant enrichment at the 0.01 level (*p* < 0.01), and *** indicates highly significant enrichment at the 0.001 level (*p* < 0.001).

To further elucidate the biological functions and key metabolic pathways associated with the differentially expressed genes (DEGs), a KEGG pathway enrichment analysis was conducted. As shown in Table 2 and Figure 5, the DEGs were primarily classified into five major categories: Metabolism, Cellular Processes, Genetic Information Processing, Organismal Systems, and Environmental Information Processing. Among these classifications, the metabolism-related pathways contained the highest number of DEGs, encompassing multiple levels from primary to secondary metabolism. Notably, the fatty acid metabolism pathway exhibited the most significant enrichment (*p* < 0.001), followed by the biosynthesis of unsaturated fatty acids pathway (*p* < 0.01). Additionally, the fatty acid biosynthesis, phosphonate and phosphinate metabolism, and longevity regulating pathways also demonstrated significant enrichment (*p* < 0.05). These enrichment results suggest that *TipE* knockdown is associated with changes in lipid homeostasis and basal metabolic processes in *M. usitatus*.

### 3.5. RT-qPCR Validation of SCD, CYP307a1, and Na_v_ Expression

**Figure 6:**
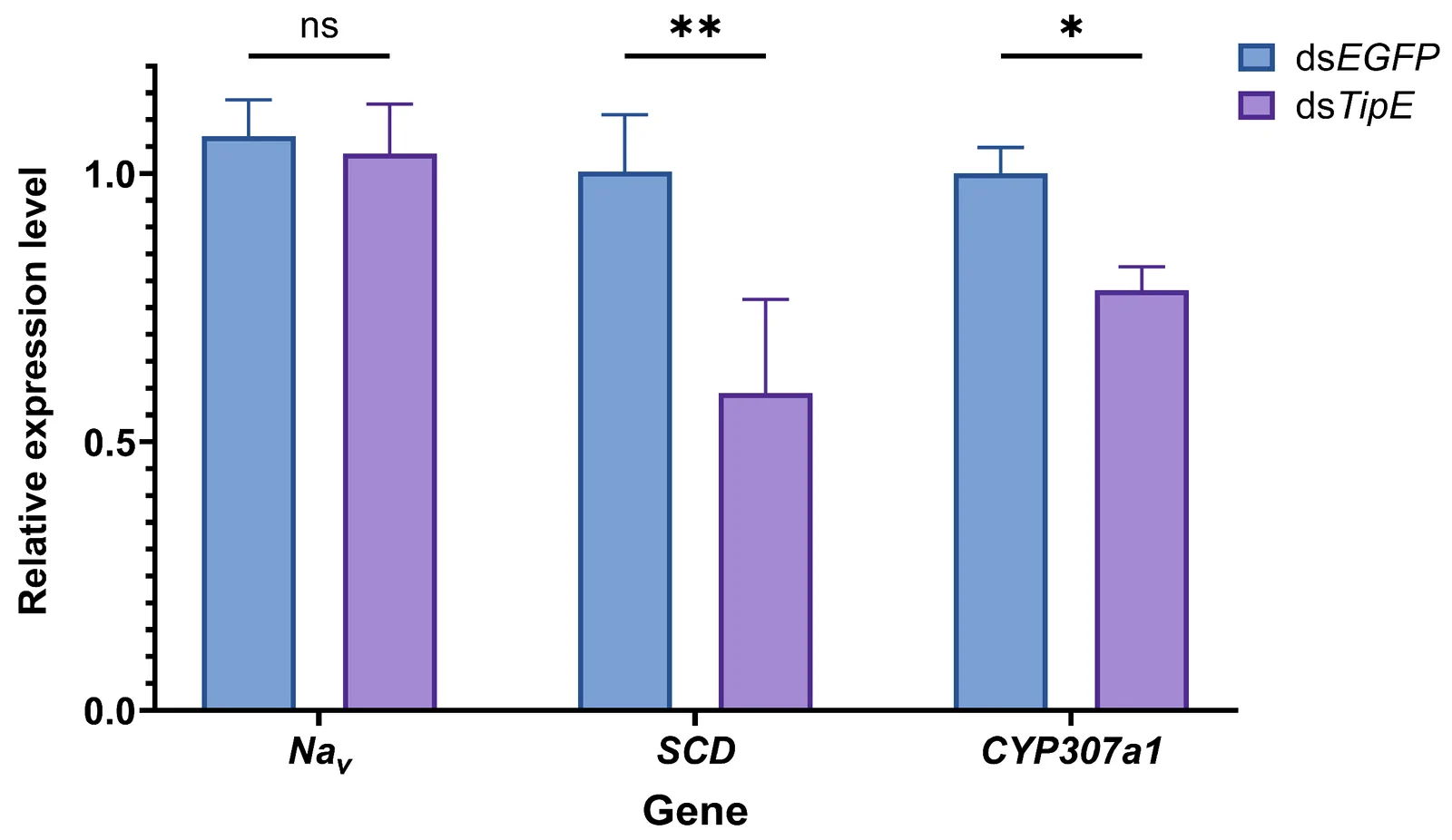
Relative expression levels of Na_v_, *SCD*, and *CYP307a1* after ds*TipE*-mediated *TipE* knockdown. For the significance analysis by Tukey’s test, * indicates a significant difference at the 0.05 level (*p* < 0.05), ** indicates a very significant difference at the 0.01 level (*p* < 0.01), and ns indicates no significant difference (*p* > 0.05).

To identify candidate genes connecting enriched metabolic pathways with strong transcript-level responses, we cross-compared genes in the enriched pathways shown in Figure 5 with the top 30 most significantly down-regulated DEGs presented in Figure 4. This analysis identified *ONE63_003354* and *ONE63_001747* as two overlapping candidate genes. Based on NCBI homology alignment and annotation, *ONE63_003354* was annotated as *SCD* (stearoyl-CoA desaturase 5-like isoform X1), a key enzyme in fatty acid desaturation and lipid homeostasis, whereas *ONE63_001747* was annotated as *CYP307a1*, an ecdysteroid-biosynthesis-related Halloween gene. Because *TipE* is an auxiliary subunit of the sodium channel, we also examined whether *TipE* knockdown altered the transcript abundance of the pore-forming Na_v_ α-subunit. RT-qPCR analysis (Figure 6) showed that Na_v_ α-subunit expression did not differ significantly between the *dsTipE* and *dsEGFP* groups. By contrast, *TipE* knockdown significantly reduced *SCD* expression by 40.90% (*p* < 0.01) and *CYP307a1* expression by 21.73% (*p* < 0.05). These results indicate that *TipE* knockdown is associated with reduced expression of two metabolism-related candidate genes, while the Na_v_ α-subunit transcript level remains stable. Thus, the increased pyrethroid-associated mortality observed after *TipE* knockdown may involve changes in metabolic or membrane-associated physiological pathways rather than a simple decrease in Na_v_ transcript abundance.

## 4. Discussion

Previous studies have shown that insect-specific *TipE* and *TipE*-homologous proteins can modulate voltage-gated sodium-channel expression, membrane localization, gating properties, and pharmacological sensitivity [13,14,15,16,17,18]. This functional background provides a basis for examining whether *TipE* contributes to pyrethroid-related responses in *M. usitatus*, a species in which pyrethroid-resistance research has mainly focused on the pore-forming Na_v_ α-subunit and kdr-associated mutations. In the present study, RNAi-mediated *TipE* knockdown increased adult mortality after exposure to λ-cyhalothrin and permethrin, suggesting that this auxiliary subunit is associated with pyrethroid-associated mortality in the tested long-term laboratory colony of *M. usitatus*. However, because sodium-channel currents and channel localization were not directly measured, the link between *TipE* knockdown and sodium-channel function should be interpreted as a working hypothesis that requires electrophysiological or cellular validation.

The optimal RNAi condition identified in this study was feeding with 400 μg/mL of ds*TipE* for 48 h, which reduced *TipE* expression by 32.55%. Following *TipE* knockdown, adult mortality increased by 8.36–15.64% after λ-cyhalothrin treatment and by 13.97–21.71% after permethrin treatment. These results indicate that *TipE* knockdown can enhance pyrethroid-associated mortality under the tested laboratory conditions. However, the knockdown efficiency was moderate, and the present bioassays were conducted under controlled laboratory conditions. Therefore, the potential use of *TipE* as an RNAi-based resistance-management target will require further validation of dsRNA delivery efficiency, target specificity, persistence, non-target risk, and field performance. Because this colony has been maintained under laboratory conditions since 2017, its current pyrethroid susceptibility may not fully represent that of field populations. Because this colony has been maintained under laboratory conditions since 2017, its current pyrethroid susceptibility may not fully represent that of field populations. Therefore, our results should be interpreted as evidence from a laboratory colony showing that *TipE* knockdown is associated with increased mortality after pyrethroid exposure, rather than as direct evidence for the field efficacy of λ-cyhalothrin or permethrin. Future studies should compare these findings with field populations or colonies with different levels of pyrethroid resistance.

Transcriptome analysis identified 205 DEGs after *TipE* knockdown, and RT-qPCR validation confirmed reduced expression of *SCD* and *CYP307a1*, whereas Na_v_ α-subunit transcript abundance did not change significantly. This pattern suggests that *TipE* knockdown may affect downstream physiological or metabolic responses without necessarily altering Na_v_ transcript levels. As an auxiliary subunit, *TipE* may influence channel function through effects on gating, trafficking, or membrane localization rather than through direct regulation of Na_v_ mRNA abundance. Functional compensation by *TipE*-homologous proteins may also contribute to the stable Na_v_ transcript level, but this possibility remains to be tested. Overall, the transcriptome results should be interpreted as evidence of associated gene-expression changes rather than proof of a direct regulatory cascade.

Cytochrome P450 monooxygenases are involved in xenobiotic metabolism, development, hormone biosynthesis, and homeostasis in insects [30,31]. *CYP307a1* is generally recognized as a Halloween gene associated with ecdysteroid biosynthesis rather than as a canonical pyrethroid-detoxification gene. Therefore, the reduced expression of *CYP307a1* after *TipE* knockdown suggests that ecdysteroid-biosynthesis-related metabolic processes may be affected, but it does not by itself demonstrate reduced pyrethroid detoxification. *SCD* is a key enzyme in lipid metabolism that converts saturated fatty acids into unsaturated fatty acids and contributes to membrane lipid homeostasis [33,34,35]. Because voltage-gated sodium channels are embedded in the lipid bilayer, changes in lipid metabolism may influence membrane properties and neural excitability. Nevertheless, direct links among *TipE* knockdown, *SCD* expression, membrane lipid composition, and pyrethroid susceptibility remain to be experimentally verified.

In summary, this study provides a working model in which *TipE* knockdown increases pyrethroid-associated mortality and is accompanied by transcriptional suppression of endocrine- and lipid-metabolism-related genes in *M. usitatus*. The value of this model is that it generates specific, testable downstream candidates rather than treating the transcriptome as a descriptive gene list. The down-regulation of *CYP307a1* and *SCD* provides candidate pathways for future study, but the causal roles of these genes in pyrethroid susceptibility remain unresolved. Further experiments, such as independent RNAi of *CYP307a1* and *SCD*, rescue assays, enzyme-activity measurements, lipidomic analysis, and electrophysiological characterization of sodium-channel function, will be needed to clarify whether these transcriptional changes directly contribute to the observed susceptibility phenotype.

## 5. Conclusions

This study provides functional evidence that the insect-specific sodium-channel auxiliary subunit *TipE* is associated with pyrethroid-associated mortality in *M. usitatus*. By integrating RNAi, insecticide bioassays, transcriptome profiling, and RT-qPCR validation, the study links an auxiliary-channel component with downstream metabolic transcriptional responses. The main conclusions are as follows: First, feeding with 400 μg/mL of ds*TipE* for 48 h reduced *TipE* expression and increased adult mortality after exposure to λ-cyhalothrin and permethrin. These results suggest that *TipE* is associated with pyrethroid-associated mortality under laboratory conditions and is a candidate gene for further functional validation. Second, transcriptome analysis and RT-qPCR validation showed that *SCD* and *CYP307a1* transcript levels were reduced following *TipE* knockdown, whereas Na_v_ α-subunit transcript abundance remained unchanged. These results suggest that the effect of *TipE* knockdown may involve endocrine- and lipid-metabolism-related transcriptional responses rather than changes in Na_v_ transcript level alone. These findings broaden the candidate target space for pyrethroid-resistance research in *M. usitatus* from Na_v_ α-subunit mutations to auxiliary-channel regulation and associated endocrine/lipid metabolic responses. Future work should determine whether *CYP307a1*, *SCD*, or other DEGs directly contribute to the increased pyrethroid-associated mortality observed after *TipE* knockdown. Field-oriented application of *dsTipE* will also require optimization of delivery systems and evaluation of stability, specificity, ecological safety, and efficacy under field conditions. Future studies should further evaluate ds*TipE*-mediated effects in field-collected *M. usitatus* populations with different pyrethroid-resistance backgrounds.

## Figures and Tables

**Table 1:** Output and quality statistics of transcriptome sequencing data of *M. usitatus*.

Sample	Raw Reads	Clean Reads	Effective Rate (%)	GC (%)	Q20 (%)	Q30 (%)
ds*EGFP*-1	50849546	49417690	97.03	43.02	96.19	93.58
ds*EGFP*-2	51094440	49447066	96.60	43.11	95.98	93.26
ds*EGFP*-3	47413624	46066108	96.98	43.02	96.23	93.66
ds*TipE*-1	45088780	43636366	96.60	42.70	95.98	93.25
ds*TipE*-2	43460744	42122906	96.75	42.85	96.04	93.36
ds*TipE*-3	41419518	40105026	96.64	42.88	96.10	93.45

**Table 2:** Statistics of KEGG pathway enrichment analysis of DEGs after *TipE* subunit silencing in *M. usitatus*.

Pathway Category	Pathway Name	Count	*p*-Value	Up Genes	Down Genes
Metabolism	Fatty acid metabolism	4	0.0006		*ONE63_003354*, *ONE63_006967*, *ONE63_002325*, *ONE63_010142*
	Biosynthesis of unsaturated fatty acids	2	0.0075		*ONE63_003354*, *ONE63_002325*
	Fatty acid biosynthesis	2	0.0184		*ONE63_006967*, *ONE63_010142*
	Phosphonate and phosphinate metabolism	1	0.0278		*ONE63_002535*
	Sphingolipid metabolism	1	0.0970	*ONE63_009610*	
	Pentose phosphate pathway	1	0.0970	*ONE63_010520*	
	Other glycan degradation	1	0.1072	*ONE63_009610*	
	Metabolic pathways	7	0.1116	*ONE63_009610*, *ONE63_010520*	*ONE63_010142*, *ONE63_006967*, *ONE63_002325*, *ONE63_002535*, *ONE63_003354*
	Insect hormone biosynthesis	1	0.1173		*ONE63_001747*
Cellular Processes	Endocytosis	2	0.1522		*ONE63_002571*, *ONE63_002684*
	Phagosome	1	0.3147		*ONE63_007899*
	Lysosome	1	0.4383	*ONE63_009610*	
Genetic Information Processing	Spliceosome	2	0.1365		*ONE63_002571*, *ONE63_002684*
	Protein processing in endoplasmic reticulum	2	0.1522		*ONE63_002571*, *ONE63_002684*
	Ubiquitin mediated proteolysis	1	0.3901		*ONE63_001113*
Organismal Systems	Longevity regulating pathway	2	0.0309		*ONE63_002571*, *ONE63_002684*
	Toll and Imd signaling pathway	1	0.2739	*ONE63_004690*	
Wnt signaling pathway	Wnt signaling pathway	1	0.3647		*ONE63_010651*

## Data Availability

The data supporting the findings of this study are not publicly available due to privacy and ethical restrictions. Requests for access may be directed to the corresponding authors and will be considered subject to applicable ethical approvals, participant consent, and data-protection requirements.
